# Personalized Approach in Transcatheter Palliation of Congenital Heart Disease with Duct-Dependent Pulmonary Circulation: Right Ventricular Outflow Tract Stenting vs. Arterial Duct Stenting

**DOI:** 10.3390/jpm14030302

**Published:** 2024-03-12

**Authors:** Silvia Teresa Scalera, Alessandra Pizzuto, Pietro Marchese, Massimiliano Cantinotti, Eliana Franchi, Chiara Marrone, Nadia Assanta, Giuseppe Santoro

**Affiliations:** Pediatric Cardiology and GUCH Unit, Heart Hospital “G. Pasquinucci”, National Research Council-Tuscany Foundation “G. Monasterio”, Via Aurelia Sud, 54100 Massa, Italy

**Keywords:** tetralogy of Fallot, pulmonary stenosis, univentricular heart, arterial duct stenting, right ventricular outflow stenting

## Abstract

Despite significant improvements in techniques, the treatment of neonates and infants with congenital heart disease resulting in duct-dependent pulmonary circulation is still significantly challenging. Despite current trends toward early primary surgical repair, temporary palliation is still necessary in those patients who are at high surgical risk for complete correction due to unfavorable clinical or anatomic characteristics. Recent advances in interventional cardiology have led to the emergence of right ventricular outflow tract and arterial duct stenting as cost-effective alternatives to surgical palliation in high-risk surgical candidates or whenever short-term pulmonary blood flow support is anticipated. This review aims to explore the evolving landscape of these transcatheter approaches, highlighting their role, efficacy and potential complications in the context of duct-dependent pulmonary circulation anatomies.

## 1. Introduction

### 1.1. Historical Perspective

Congenital heart diseases with duct-dependent pulmonary blood circulation comprise a wide spectrum of anomalies characterized by severe pulmonary hypoperfusion at the time of arterial duct (AD) closure and burdened by high morbidity and mortality at neonatal age. The most frequent cardiac malformations are critical pulmonary stenosis (PS)/pulmonary atresia with intact ventricular septum (PA-IVS) and tetralogy of Fallot (TOF)/pulmonary atresia with ventricular septal defect (PA-VSD). Less frequently diagnosed are complex congenital heart malformations with univentricular physiology and pulmonary stenosis/atresia destined for Fontan-type repair. In all of them, pulmonary circulation may be critically dependent on the AD. Thus, spontaneous AD closure during the perinatal period may result in severe pulmonary hypoperfusion and profound desaturation, with metabolic changes potentially leading to patient death.

In the case of TOF and well-developed confluent pulmonary arteries, the majority of patients typically undergo uncomplicated elective primary repair within the first year of life with excellent results [1,2]. Although neonatal repair of this malformation has been routinely performed in some centers, delayed repair beyond the first 3–6 months of life has been found to be cost-effective in terms of early post-operative course and overall survival outcome [1].

However, some neonates who are critically dependent on the accessory sources of pulmonary blood flow cannot wait for complete primary repair and are candidates for the palliative procedure. These patients are dependent on continuous prostaglandin infusion due to precarious or antegrade-lacking pulmonary blood flow as a consequence of an unfavorable RVOT anatomy—such as infundibular or valvar stenosis or atresia—and/or pulmonary arterial tree, as in the case of hypoplastic vessels and multiple aorto-pulmonary collateral arteries (MAPCAs). Additionally, neonatal comorbidities, such as prematurity, low weight, infections, neurological injuries and other conditions requiring non-cardiac surgery, often require longer time to stabilize or have to be fixed before cardiac repair. All these patients greatly benefit from a stabilized source of pulmonary blood flow in view of subsequent lower risk surgical repair. Continuous intravenous infusion of prostaglandin E achieves this aim for a relatively short period by maintaining AD patency, but medium-term palliation—providing a stable and more durable pulmonary blood flow source—is often necessary in most cases. In addition, long-term prognosis may be particularly poor in neonates with extra-cardiac comorbidities, even if primary repair can be feasible, and it has been associated with increased morbidity in large surgical series [1,3].

In this context, early palliation is suggested and performed worldwide via aorto-pulmonary shunt, which is commonly used as a bridge to complete repair [4]. The decision-making process regarding primary repair is nuanced, with no clear-cut boundaries. Centers achieving favorable outcomes with early infant repair have identified specific risk factors, including low weight, severe cyanosis, pulmonary atresia (as opposed to stenosis), hypoplastic pulmonary arteries and extra-cardiac comorbidities, which are associated with higher mortality and the likelihood of re-intervention [5]. In addition, the use of mBT shunt in premature infants with low weight and hypoplastic pulmonary arteries is associated with increased complications, such as pulmonary artery stenosis and pulmonary over-circulation [6].

In fact, the morbidity and mortality from surgical shunt are reported as high as 15% in large series [4,5,6], thus emphasizing that despite its perceived safety, the modified Blalock–Taussig shunt is associated with high morbidity and mortality rate. The most frequent potential complications are phrenic or vagal nerve paralysis, chylothorax, differential growth of pulmonary artery branches, distortion of pulmonary arteries, pulmonary hypertension, surgical adhesions, early shunt occlusion and/or stenosis.

In this setting, the transcatheter approach has been evaluated as a cost-effective alternative in order to tailor the amount of pulmonary blood flow to patient size and clinical conditions. The tools potentially available to achieve this aim are RVOT stenting or AD stabilization. Both of them showed high efficacy in improving arterial oxygen saturation and promoting balanced pulmonary artery growth [7,8,9,10,11,12,13,14,15,16,17,18,19].

A percutaneous increase in pulmonary blood flow, achieved through RVOT or AD stenting, was first reported by Gibbs et al. [8], but the initial results were not encouraging due to the primitive technology and technique. However, this approach was re-introduced from 2010 onwards as a cost-effective modality of palliation thanks to the significant advances in techniques and materials, and in a few years, it became an effective alternative to surgical systemic-to-pulmonary artery shunt in neonates who are unsuitable for primary repair or in whom spontaneous improvement of oxygen saturation can be anticipated at the time of spontaneous pulmonary vascular resistance drop.

RVOT stenting is technically less demanding as compared to AD stenting and has been shown to provide good palliation with low complications. In addition, it can be the sole option for older patients in whom the AD is completely closed beyond the first weeks of life.

Conversely, in newborns with severe TOF with critical RVOT obstruction or atresia, AD stenting is the sole alternative to surgical aorto-pulmonary shunt. By enabling the fine-tuning of shunt magnitude for the individual patient, AD stenting seems to have more favorable effects—as compared to surgery—on the development of the pulmonary vascular tree due to even distribution of the pulmonary blood flow [9].

The aim of this review is to highlight the potential and the advantages of percutaneous palliation of cardiac malformations with pulmonary duct-dependent circulation, as well as to support pediatric cardiologists in selecting the best option for the individual patient, given the considerably greater patient-tailored approach.

### 1.2. Indication and Contraindications for RVOT Stenting

**Figure 1:**
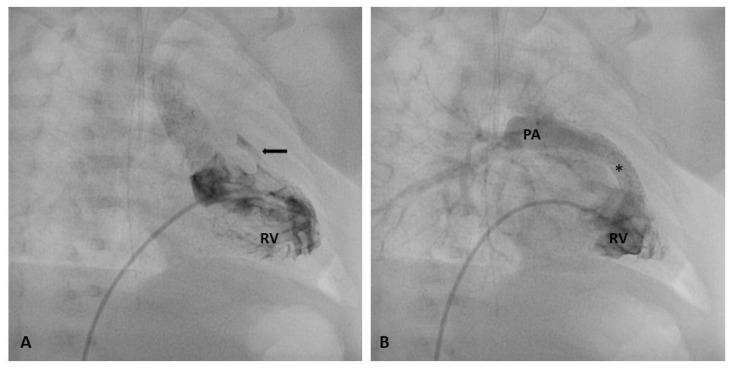
Stenting of severely stenotic RVOT (**arrow**). Baseline right ventricular angiography showing an almost completely atretic RVOT before (**A**) and after stent implantation (**B**). At final angiography, a significant improvement in pulmonary artery flow is clearly imaged. * stent; Abbreviations: RV, right ventricle; PA, pulmonary artery.

Over time, several groups have consistently suggested RVOT stenting as initial palliation for symptomatic patients with Fallot-type lesions (Figure 1).

At the beginning, RVOT stenting was introduced as an alternative to primary palliative or corrective surgery in high-risk infants with underdeveloped PAs and hyper-cyanotic spells during the neonatal period in whom surgical palliation could not be performed at a reasonable risk [9,10,11,12]. This option increases pulmonary blood flow, improving systemic saturation and hemodynamic conditions. Notably, different from surgical shunt, RVOT stenting results in a balanced pulmonary blood flow and thus promotes a homogeneous growth of pulmonary arteries [12].

Although the choice between RVOT stenting and surgical palliation strongly depends on the local policy of any individual center and the expertise of both the interventional cardiologist and pediatric cardiac surgeon, this percutaneous approach is nowadays considered as generally safer and more physiologic than mBTS. The following may be used as criteria for guiding inclusion into a stenting program:

1. Patients with a high-risk profile for conventional surgical palliation: low-birth-weight neonate with or without any significant comorbidity or with unfavorable anatomic arrangement of pulmonary arteries, such as pulmonary artery discontinuity or hypoplasia.
2. Patients at low risk for surgical palliation but with an anticipated need for short-term support for pulmonary circulation due to early surgical repair.

RVOT stenting might be challenging in anatomical limitations, such as complex RVOT anatomy, pulmonary infundibular/valve atresia or severe pulmonary valvular hypoplasia. In these settings, a hybrid approach with stent implantation under direct vision via surgical sternotomy may be a cost-effective alternative to the standard percutaneous option. Conversely, the coronary artery pattern does not seem to significantly impact the procedural results or outcomes.

This procedure has gained prominence in the last decade as an alternative palliative option in infants with cyanotic heart defects—particularly those with decreased pulmonary blood flow—with fewer post-procedural complications and better physiological pulmonary artery growth, which in turn can significantly impact the success of final surgical repair. RVOT stenting is typically performed in the early newborn period, although it is not uncommon for it to be conducted at a later age for various reasons.

The indications for RVOT stenting include neonates with risk factors such as prematurity, low birth weight, infections and other extra-cardiac conditions requiring surgery. Additionally, it serves as a bail-out procedure in emergent conditions with uncontrolled cyanotic spells. Comorbidities can complicate or delay primary cardiac repair, making RVOT stenting a valuable option.

### 1.3. Indication and Contraindications for AD Stenting

**Figure 2:**
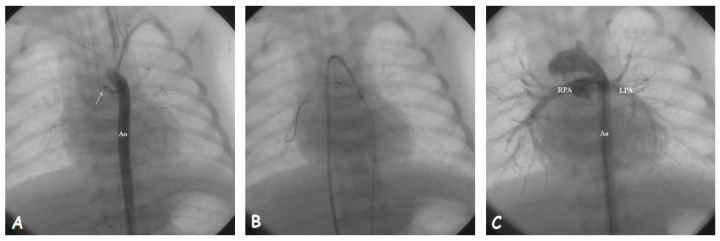
Stenting of a closed arterial duct (**arrow**). (**A**). Aortic angiography showing the proximal stump of arterial duct arising from the underside of aortic arch. (**B**). The guidewire is passed through the duct after local infusion of prostaglandin E and anchored into the right lower lobe pulmonary artery. The stent is being deployed under the guide of aortic angiographies performed from the venous access. (**C**). Final angiographic result after stent implantation; Abbreviations: Ao, aorta; LPA, left pulmonary artery; RPA, right pulmonary artery.

AD stenting represents an appealing mini-invasive alternative to surgical aorto-pulmonary shunt (Figure 2). Early bending or thrombosis of stented arterial duct/surgical conduit is reported in 5–20% of patients, with higher rates after surgical palliation, which results in higher mortality and morbidity in comparative series. At mid-term follow-up, either procedure shows a gradual decline in arterial oxygen saturation due to tissue growth within the ductal stents (intimal ingrowth) or the development of fibro-intimal peel or clots within the surgical shunt. However, surgical shunt warrants longer palliation with respect to DS, which shows a longevity period of only 6–12 months. In fact, the rate of re-intervention is significantly higher following AD than surgical palliation, albeit, in most cases, this is due to stent re-dilatation of under-sized stents implanted in low-weight neonates. This discrepancy in palliation duration might influence the selection of patients for either procedure [13].

Over time, several small series showed excellent results of AD stenting employed as a palliation procedure for pulmonary duct-dependent cardiac malformations. The initial experiences over the last few decades [14] have been encouraging in small series and, over time, larger series, in even more complex anatomic/clinical settings [14,15,16,17,18], clearly showing the superiority of this approach with respect to surgical palliation.

Alwi et al. [19] showed that arterial duct stenting is a favorable, cost-effective alternative to surgical shunt in most patients with duct-dependent circulation, with the only absolute contraindication to this approach being represented by pre-existing branch pulmonary stenosis.

Gewillig et al. [9] demonstrated that, using the current technology, complete stenting of a short and straight duct is a secure and efficient method of palliation, allowing sufficient growth of the pulmonary arteries.

Santoro et al. [20] reported a 93.3% success rate in ductal stenting procedures, with a 17.6% complication rate and 3.6% in-hospital mortality. Elective Blalock–Taussig shunt procedure was required in 13.5% of cases. Pre-surgical catheterization showed significant and balanced pulmonary artery growth, especially in patients with severely hypoplastic pulmonary arteries at duct stabilization. The study concluded that ductal stenting is feasible and effective in promoting balanced pulmonary artery growth, particularly in patients with hypoplastic main pulmonary arteries.

Over time, large multi-center series have clearly and definitively demonstrated the superiority of AD stenting over surgical shunt in terms of complications, length of hospital stay and mortality and costs [15,21,22,23,24,25,26,27].

According to the 2011 AHA Scientific Statement [25], AD stenting deployed for provision of additional pulmonary blood flow can be used in the following scenarios:

1. Patients with a high-risk profile for surgery, having an anatomically suitable ductus arteriosus and more than one source of pulmonary blood flow.
2. Patients at low risk for surgical palliation but with an anticipated need for short-term support for pulmonary circulation due to early surgical repair or while waiting for the evolution of clinical scenarios.

However, AD stenting in infants with cyanotic CHD and pulmonary artery stenosis near ductal insertion is still not recommended [26]. In addition, the procedure may be complicated depending on the anatomy of the duct. The anatomy of AD in infants with duct-dependent pulmonary blood flow (PBF) varies widely, influencing the outcomes of the procedure. Qureshi et al. [11] classified the ADs into three tortuosity index (TI) groups (Type I, II, III), and hence, into subtypes based on the ductal origin from the systemic vessels. On the basis of their angiographic appearance, the curvature index (CI) is calculated, with higher CI being associated with increased duct curvature, correlated with unplanned interventions and pulmonary branch jailing. The findings underscore the importance of considering AD anatomy and curvature in planning interventions for improved outcomes.

### 1.4. Pathophysiologic Differences in Transcatheter Palliation Approaches

Either RVOT stenting or AD stenting enables a significant and balanced increase in systemic arterial saturation by mimicking the physiologic pattern of pulmonary blood flow. However, some differences should be reported in view of a patient-tailored approach.

RVOT stenting results only in systolic—although well-distributed—pulmonary blood flow. In addition, the stent size strongly depends on RVOT anatomy, as well as the size of the pulmonary valve annulus. Finally, the rhythmic contraction of the right ventricular infundibulum often results in stent strut fractures and may cause early recurrence of RVOT obstruction.

AD stenting results in a continuous but not always balanced flow due to the origin and orientation of the duct. This potential flow streaming might worsen any potential original size discrepancy between the pulmonary arteries found at the time of palliation. However, despite this potential pathologic blood flow streaming and the occurrence of pulmonary stenosis at the site of duct implantation, the growth of the pulmonary arteries following duct stabilization is good and significantly more balanced than after mBT shunt [23,26]. In addition, the size of the stent potentially used to stabilize the AD may be more precisely set and tailored to patient size and the anticipated time of palliation with respect to a surgical shunt, either in the case of normal size of pulmonary arteries or in the case of hypoplastic or discontinuous pulmonary arteries [13,14,15,21,28]. In fact, in low-weight neonates, it is possible to under-size the stent diameter and further re-dilate it at mid-term follow-up during patient growth [14]. This approach significantly decreases the risk of pulmonary over-circulation and congestive heart failure, which often complicate the early post-operative course of these frail patients. However, pulmonary artery stenosis at the site of stent implantation is a frequent complication of this approach, although no significant differences in surgical technique and complications have been reported in the literature.

In conclusion, significant differences in the pathophysiology and size of the potentially used stent should be accounted for whenever transcatheter palliation is chosen with respect to surgical options.

### 1.5. Procedural Technique: RVOT Stenting

The procedure, as outlined by Quandt and Stumper et al. [29,30], involves a thorough pre-procedure evaluation and is typically carried out under general anesthesia and mechanical ventilation. This approach is preferred due to the typically hypoxic and frail condition of the neonate, who can rapidly deteriorate during the procedure. Prostaglandin infusions are continued, and all necessary emergency drugs are readily available. Access is commonly established via the right femoral vein, although the internal jugular venous approach may sometimes be preferred, especially in smaller patients. Right ventricular angiogram is performed through a diagnostic catheter placed within the apex of the right ventricle, in the right oblique view or different specific projections, in order to detail the RVOT anatomy and size, the site of stenosis (infundibular, valvular, multiple), the pulmonary valve annulus and the anatomy as well as the size of pulmonary arteries. The selection of stent type and size is guided by the size of the patient, the anatomy of the outflow tract, as well as the anticipated duration of palliation. For short-term palliation in smaller neonates, a coronary stent is preferred, while older patients (infants who cannot undergo the repair procedure due to comorbidities or high surgical risk) may receive a bare metal peripheral vascular stent to achieve a medium–longer term palliation. The use of balloon-mounted stents is preferred, although thicker wires may be required, potentially causing RVOT spasm and desaturation.

Following stent selection, an appropriate delivery sheath or guide catheter is used, and the pre-mounted stent is advanced over the wire within the delivery sheath and fully uncovered after confirming the position with test angiograms. The balloon is then inflated, and the stent position is angiographically checked. The entire procedure is meticulously imaged to assess stent position, branch pulmonary arteries’ opacification and pulmonary valve movements.

The complications include malposition or migration of the stent, tricuspid valve leaflet entrapment and malfunction. The use of a long delivery sheath is employed to minimize these risks. Other potential complications involve balloon rupture, dissection, stent-induced pulmonary edema, arrhythmias and tricuspid valve injury.

Despite the challenges and technical difficulty associated with the procedure, RVOT stenting has become a safer alternative, particularly in centers with early-stage cardiac programs.

### 1.6. Procedural Technique: AD Stenting

Arterial duct stenting is often performed under general anesthesia. Providing adequate pre-load, contractility (via inotropes/vasopressors if needed), preventing an increase in pulmonary vascular resistance (PVR) by avoiding hypoxia, hypercarbia and acidosis, modulating systemic vascular resistance (SVR) and maintaining normothermia are the key factors during the procedure. It is crucial to have surgical support and quick access to venous-arterial extra-corporeal membrane oxygenator support (VA-ECMO) in case of any severe complications or hemodynamic instability. In the case of infants under 2 kg or with other medical issues, the decision-making process should include the ability to place the infant on VA-ECMO if they experience hemodynamic instability, which cannot be managed with medication or rapid stent deployment. Prostaglandin infusion should be stopped a few hours before the procedure in order to increase AD vasoreactivity and make stent-grasping inside the duct easier. However, it is sometimes necessary to have prostaglandin available for local infusion in case of sudden duct spasm at the time of stent positioning. Detailed imaging of AD via echocardiography and 3D CT scan reconstruction [30] is mandatory for selecting the best vascular access and minimizing the procedural risk and length. Different access sites can be used on the basis of duct origin and tortuosity, with the aim of being as straight and close as possible to the duct. In case of extreme duct tortuosity, vascular access should be the closest possible to the duct in order to angiographically guide stent positioning and deployment. Alternatively, a second vascular access could be suggested in order to guide the procedure via serial angiographies. In the vast majority of cases, femoral artery, femoral vein, common carotid artery and axillary artery may be used. Less frequently, the procedure may be performed via umbilical artery or umbilical vein. AD arising from the descending aorta can be easily approached via the femoral artery, while those originating from the epi-aortic vessels may be probed and stabilized from the femoral artery, carotid artery or axillary artery. The common carotid or axillary artery approach is more common in ducts with higher tortuosity index and in smaller patients, since it allows angiographic evaluation of positioning and deployment of the stent via multiple angiographies from the introducer sheath. Finally, in case of vertical duct or anomalous ducts originating from the ascending aorta, carotid artery access is preferable. However, in case of cardiac malformations with ventricular septal defect, venous access may also provide access to the duct. Angiograms are taken to analyze the ductus and prepare the equipment for stenting. The stent must cover the entire AD to prevent re-stenosis. The size of the stent strongly depends on the weight of the patient. Stent size should be 3.5–4 mm in neonates >3 kg, 3–3.5 in neonates <3 kg and 2.5–3 mm in neonates <2 kg. However, stent size should always be determined based on clinical conditions, the hemodynamic relevance of potential accessory pulmonary flow sources, as well as the expected time of palliation. AD probing is performed using a 0.014″ floppy guidewire, and a microcatheter may be used in selected cases. A stiff wire should be avoided in order to prevent duct spasm during guidewire manipulation. The stent can be delivered with or without a guiding catheter and should mandatorily cover the whole length of the duct, minimizing any protrusion into the aortic or pulmonary ends. In case of pulmonary artery stenosis at the site of duct insertion, it could be preferable to enter the stenotic pulmonary artery branch or jail the stenotic vessel and re-dilate it passing through the stent side cell.

The procedural complications of AD stenting include injury to the blood vessels, spasm or dissection of the duct during the probing procedure, stent migration and acute thrombosis within the stent. All of them can result in cyanosis and the risk of significant hemodynamic compromise (potentially requiring emergency surgical intervention). Proper sizing during stent placement and careful withdrawal of the balloon and wire can prevent stent migration. Adequate anticoagulation with heparin is essential to prevent stent thrombosis, and angioplasty (with anticoagulation or thrombolysis if feasible) may be necessary in case of acute stent thrombosis.

## 2. Discussion

Neonates and infants with cyanotic congenital heart disease and duct-dependent pulmonary blood flow need a palliative procedure before complete surgical repair. Over time, some non-surgical approaches have been proposed to avoid surgical palliation and its inherent risks. Two different transcatheter approaches have been tested to increase pulmonary blood flow, i.e., right ventricular outflow stenting and arterial duct stenting, with different pathophysiology, technical advantages and drawbacks, as well as anatomic settings. The overall knowledge of these data may be crucial in deciding on the best approach for the patient as part of a patient-tailored therapy. Percutaneous palliation through RVOT and ductal stenting provides less durable palliation than a conventional surgical shunt due to tissue reaction and fibrous tissue ingrowth, ductal tissue prolapses through the stent struts and intra-stent endothelial hyperplasia. The use of newer technologies, such as drug-eluting stents, could overcome this shortfall and improve the long-term patency of the stented portions. In addition, RVOT stenting often leaves metallic remnants embedded into the RVOT myocardium, which can be incompletely removed at the time of surgical repair. It is still unknown whether these retained stent fragments may be a focus for future ventricular arrhythmias or infections. Although removing the stent may lengthen surgical repair, the cardio-pulmonary bypass time seems to be similar to trans-annular patch repairs of non-stented patients. Importantly, most studies report that after the palliation period, there are no differences in the type of surgical repair, either in biventricular or single-ventricle repair [31,32,33,34]. Finally, the overall mortality rate during palliation with RVOT stenting appears to be similar to that after mBT shunt.

Despite lower durability than the conventional surgical shunt, percutaneous palliation results in a greater rise in oxygen saturation and promotes better and more constant symmetrical pulmonary arterial growth than mBT shunt palliation. This is possibly due to a balanced flow to the main pulmonary artery branches compared with the unfavorable graft geometry of a surgical shunt, which usually causes overgrowth of the ipsilateral pulmonary artery and lower development of the contralateral pulmonary artery. In recent years, ductal stenting has increasingly been considered safer and more effective than surgical palliation, particularly in high-risk patients. This is primarily because it allows for the customization of shunt size and adaptation to the patient’s size and pulmonary anatomy. Moreover, the relatively shorter durability of a stented duct makes it suitable as a temporary solution, providing support for pulmonary blood flow until spontaneous improvement occurs.

By shaping the stent according to the size and angle of the main pulmonary arteries, it is theoretically possible to achieve a more even distribution of pulmonary blood flow, promoting uniform vascular development. At mid-term follow-up, the stented duct resulted in similar systemic oxygen saturation levels despite being dilated to a smaller diameter than a conventional mBT shunt.

When ductal stenting is performed, a high rate of pulmonary artery stenosis at the site of stent insertion is described in a high percentage of patients. This complication might possibly be due to involution of the ductal tissue within the PA confluence and is easily addressed with patch augmentation at the time of repair, without significant impact on surgical morbidity and mortality.

## 3. Conclusions

The percutaneous option is a technically feasible, well-tolerated and effective palliation in duct-dependent pulmonary circulation palliation. It can effectively improve oxygen saturation and promote pulmonary arterial growth, thus lowering the risk of surgical repair. It is indicated either in neonates with a high risk for surgery or in low-risk patients who need short-/mid-term pulmonary blood flow support (less than 6 months) due to early surgical repair or in whom spontaneous improvement is anticipated (i.e., pulmonary stenosis/atresia submitted to effective percutaneous dilatation). In addition, this approach promotes a significant and more balanced growth of the pulmonary artery as compared to the mBT shunt, presumably due to a more uniform pulmonary blood flow. Although less durable than surgical palliation, either RVOT stenting or AD stenting shows comparable clinical sequelae and early mortality rates, which are significantly better than those resulting from surgical palliation [13,22,32,33]. Although local policy and interventional cardiology preference and expertise can favor both approaches, AD stenting should be preferred in case of anticipated spontaneous improvement or the need for further procedure using larger stents at patient growth, while RVOT stenting should be selected in case of early corrective surgery or whenever duct anatomy is challenging. Finally, both options might be proposed in sequence over time in a rare case, where it is mandatory to prolong the lifespan of the stented tracts.

## Data Availability

No data are reported in this paper.
